# Genome-Wide Analysis of Genes Encoding Methionine-Rich Proteins in* Arabidopsis* and Soybean Suggesting Their Roles in the Adaptation of Plants to Abiotic Stress

**DOI:** 10.1155/2016/5427062

**Published:** 2016-08-18

**Authors:** Ha Duc Chu, Quynh Ngoc Le, Huy Quang Nguyen, Dung Tien Le

**Affiliations:** ^1^National Key Laboratory of Plant and Cell Technology, Agricultural Genetics Institute, Vietnam Academy of Agricultural Sciences, Pham Van Dong Road, Hanoi, Vietnam; ^2^Department of Biochemistry and Plant Physiology, Faculty of Biology, VNU-University of Science, Vietnam National University-Hanoi, Nguyen Trai Street, Hanoi, Vietnam

## Abstract

Oxidation and reduction of methionine (Met) play important roles in scavenging reactive oxygen species (ROS) and signaling in living organisms. To understand the impacts of Met oxidation and reduction in plants during stress, we surveyed the genomes of* Arabidopsis* and soybean (*Glycine max* L.) for genes encoding Met-rich proteins (MRPs). We found 121 and 213 genes encoding MRPs in* Arabidopsis* and soybean, respectively. Gene annotation indicated that those with known function are involved in vital cellular processes such as transcriptional control, calcium signaling, protein modification, and metal transport. Next, we analyzed the transcript levels of MRP-coding genes under normal and stress conditions. We found that 57 AtMRPs were responsive either to drought or to high salinity stress in* Arabidopsis*; 35 GmMRPs were responsive to drought in the leaf of late vegetative or early reproductive stages of soybean. Among the MRP genes with a known function, the majority of the abiotic stress-responsive genes are involved in transcription control and calcium signaling. Finally,* Arabidopsis* plant which overexpressed an MRP-coding gene, whose transcripts were downregulated by abiotic stress, was more sensitive to paraquat than the control. Taken together, our report indicates that MRPs participate in various vital processes of plants under normal and stress conditions.

## 1. Introduction

Under elevated ROS levels, free and protein-based Met are converted to methionine sulfoxide (MetO) which occurs in a diastereomeric mixture of methionine-*S*-sulfoxide (Met-*S*-O) and methionine-*R*-sulfoxide (Met-*R*-O) [1]. Oxidation of Met was reported to occur in various signaling proteins, thereby modulating their functions [1–9]. For example, calmodulin (CAM), a versatile protein involved in various signaling pathways, including ROS homeostasis in* Arabidopsis* [10], is well known to have its methionine residue oxidation linked to protein dysfunction [11] and loss of protein stability [12]. Nevertheless, efforts to systematically identify all the proteins whose methionine residues are susceptible to oxidation yielded limited results due to the lack of viable tools, including an antibody specific to methionine sulfoxide [13–16]. Recently, Tarrago and colleagues proposed an affinity chromatography approach employing methionine sulfoxide reductase (MSR) to catch interacting partners. Using AtMSRB1 as bait, the authors isolated 24 interacting partners functioning in photosynthesis, translation, and protection against oxidative stress from* Arabidopsis*. The authors found a preference of proteins with higher Met content to bind to the bait, becoming isolated by this approach [17]. Quite recently, Jacques et al. used a newly developed technique called COFRADIC [18] for proteome-wide identification of Met oxidation sites in* Arabidopsis* proteins. Their work revealed 500 sites of Met oxidation in 400 proteins of the plant [19].

Organisms evolved two enzyme families to repair oxidized Met in proteins: methionine-*S*-sulfoxide reductase (MSRA) to reduce Met-*S*-O and methionine-*R*-sulfoxide reductase (MSRB) to reduce Met-*R*-O.* In vivo* modulation of MSR activities has been reported in yeast [20, 21], fruit flies [22], and mammals [23], which in turn affected their tolerance to oxidative stress and lifespan. In plants, Romero et al. demonstrated the role of* Arabidopsis* plastidial MSRAs in the defense against oxidative stress [24]. In another study, transgenic tomato overexpressing pepper* MSR* gene* CaMSRB2* was found to protect against oxidative stress and* Phytophthora* pathogen infection [25]. Very recently, the role of* Arabidopsis* cytosolic AtMSRB7 and AtMSRB8 in conferring tolerance to oxidative stress was also demonstrated [26], whereas overexpression of AtMSRB1 and AtMSRB2 in plastids did not improve tolerance to high light stress [27].

A number of studies have documented the role of enhancing expression of MSR-coding gene(s) in conferring stress tolerance to plants, but still little is known about the MSR targets which provide such tolerance. A notable study by Laugier et al. provided indirect evidence that* Arabidopsis* plastidial MSRBs confer tolerance to high light stress by acting on cpSRP43 and cpSRP54, thereby maintaining the integrity of the photosystem antenna under environmental constraints [28]. Another recent study by Lee and colleagues on* Arabidopsis* cytosolic MSRB indicated that the enzyme conferred stress tolerance to the plants by acting on two glutathione transferases, GSTF2 and GSTF3 [29]. This study also suggested a list of potential substrates of AtMSRB7. Despite the fact that oxidation and reduction of Met residues in CAM and other calcium signaling proteins were experimentally verified to be involved in regulating the protein's functions [2–4, 11, 12], they were not found among the potential candidates acquired by either the affinity chromatography approach or approaches that employed mass spectroscopy [17, 19, 29]. This line of evidence offers opportunity to argue that either the current approaches for proteome-wide identification of MSR targets pose technical limitations or the oxidation and reduction of Met in many proteins like CAM happen transiently, such that these techniques were unable to help in identifying them. To provide a complementary approach to identify possible targets of MSR in plants, in this work, we surveyed genomes of two dicots,* Arabidopsis* and soybean, to obtain polypeptides of more than 95 residues in length with more than 6% of Met in their sequences. We analyzed these genes in terms of functions, transcriptional responsiveness to stresses, and the conservation of Met residues in HMM profiles (a hidden Markov model-based profile of amino acid residues in a protein domain). A list of genes transcriptionally responsive to stresses with an HMM profile containing highly conserved Met is provided for experimental confirmation by the research community.

## 2. Materials and Methods

### 2.1. Materials

Unless otherwise stated,* Arabidopsis thaliana* studied in this work is Columbia ecotype and soybean is of* Williams 82* cultivar.* Arabidopsis* seed overexpressing MRP gene(s) was obtained from* Arabidopsis* FOX line library (RIKEN BioResource Center, Tsukuba, Japan). Briefly, full length cDNA of* Arabidopsis* were placed under the control of 35S promoter and then inserted into a hygromycin-resistant plasmid. The plasmids were then transformed into* Arabidopsis* plants using flower-dipping technique. The development of FOX line library and the line carrying At3g55240 were previously reported [30].

### 2.2. Growth of* Arabidopsis* and Stress Treatment

*Arabidopsis* seeds were germinated on 0.5x MS media with or without antibiotics. At 2 weeks old the plants were transferred to soil and allowed to grow at 24 ± 2°C with 16-hour lighting. For paraquat leaf disc assay, 3-week-old rosette leaves were excised and placed on paraquat solutions of various concentrations; after keeping in the dark for 1 hour, the plates were kept at 24°C for 24 hours. The experiments were done in triplicate; each replicate consists of 3 plants.

### 2.3. Computational Analyses

Protein sequences were downloaded from the PHYTOZOME database (http://www.phytozome.net/) and searched for proteins of 95 residues or more whose sequences contain 6% or more Met by using a java script. The cutoff value for protein length was chosen after consulting reports on the distribution of protein sizes [31, 32]. Genes satisfying these conditions were named Met-rich proteins (MRP):* AtMRPs* for genes from* Arabidopsis* and* GmMRPs* for genes from soybean. Genes encoding MRPs were classified into functional categories using MAPMAN [33]. The PFAM database (http://pfam.xfam.org/) was used to search for HMM profiles as well as possible protein domains.

To obtain transcription levels, microarray data from previous studies were mined, including datasets for* Arabidopsis* under drought and salinity stress [34, 35] and soybean under experimental drought conditions [36]. In that study, for drought treatment of* Arabidopsis*, 2-week-old plants were transferred to soil and allowed to grow for one more week; the 3-week-old plants were then withheld from watering for 10 days. After the tenth day, rosette leaves were collected from both well-watered and drought-stressed plants, frozen in liquid nitrogen, and stored at −80°C until RNA extraction. For high salinity treatment, 10-day-old plants grown on GM media were transferred onto 0.5x MS plates without sucrose, containing either 0 mM (untreated) or 200 mM NaCl and maintained for a period of 24 h. Samples were collected in three biological replicates, frozen in liquid nitrogen, and stored at −80°C until used for RNA extraction. The drought treatment of soybean plants and data acquisition was described previously [36]. Data analyses were performed with functions integrated in MS EXCEL.

## 3. Results and Discussions

### 3.1. Occurrence of Genes Encoding MRPs in* Arabidopsis* and Soybean

**Figure 1:**
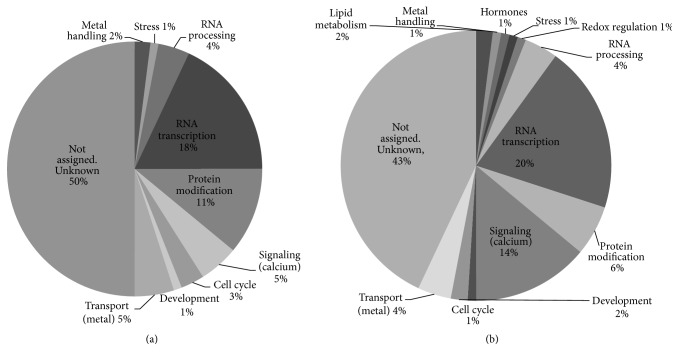
Distribution of genes encoding MRPs into various biological processes in* Arabidopsis* (a) and soybean (b).

An exhaustive search of genes encoding proteins longer than 95 residues and containing 6% Met or more resulted in 121 and 213 genes from* Arabidopsis* and soybean genomes, respectively. Functions of about 50% of those genes were not known. RNA transcription, protein modification, and calcium signaling were the three major functional categories of the MRPs analyzed (Figure 1), indicating the important roles of MRP-coding genes in overall cellular function. Smaller categories include RNA processing and metal transport. Specifically, there were 20 and 44 MRP-coding genes functioning in RNA transcription in* Arabidopsis* and soybean, respectively, making it the most abundant category. The second most abundant category was protein modification, of which 12 and 15 genes were found in* Arabidopsis* and soybean, respectively. There were 6 MRP-coding genes of* Arabidopsis* functioning in calcium signaling, whereas 31 MRP-coding genes found in soybean genome belonged to this category. Among MRP-coding genes identified, soybean has 10 genes distributed in 4 unique categories that were not presented in* Arabidopsis*, namely, lipid metabolism (4 genes), amino acid metabolism (2 genes), hormones (2 genes), and redox regulation (2 genes).

### 3.2. Stress-Responsive MRP-Coding Genes in* Arabidopsis*

To obtain MRP-coding genes transcriptionally responsive to abiotic stresses, our published microarray data of drought and high salinity treatments of wild type* Arabidopsis* [34, 35] were analyzed and data mining was performed. Of the 121* Arabidopsis* genes, expression data of 108 genes were available (Table S1 in the Supplementary Material available online at http://dx.doi.org/10.1155/2016/5427062). Further analyses indicated that 23 and 16 genes were induced and repressed more than 2-fold, respectively, by drought treatment. Under treatment by high salinity, 11 and 17 genes were induced and repressed more than 2-fold, respectively. Among drought- and salt-responsive MRP-coding genes, several genes were previously confirmed to be stress-inducible, of which the expressions of the 10 genes were responsive to both drought and high salinity (Table 1). Most of the genes were responsive to both stressors' code for plant-specific proteins. AT4G33467, encoding an unknown protein, was the most induced gene. Its transcript level was upregulated more than 330-fold by drought. Among stress-repressed genes, AT3G55240 was the most downregulated. Its transcript level was repressed by 60- and 26-fold under drought and high salinity, respectively. The function of this gene is not yet known; however, it was reported that overexpression of this gene in* Arabidopsis* led to the phenotype “pseudo-etiolation in light” [30].

### 3.3. Stress-Responsive MRP-Coding Genes in Soybean

To identify stress-responsive genes among MRP-coding genes, we performed data mining with the microarray experiments conducted earlier. In these experiments, drought treatments were carried out by withholding water. Leaves of V6 (vegetative) and R2 (reproductive) stages were collected and microarray analyses performed as reported [36]. Transcript levels of all 213 MRP-coding genes were obtained, of which 11 were repressed and 12 genes were induced under drought in V6* trifolia*. In reproductive leaves (R2* trifolia*), drought treatment induced 24 genes, whereas only 6 genes were repressed (Table S2). A total of 13 MRP-coding genes were found to be transcriptionally responsive to drought in both vegetative and reproductive-stage leaves (Table 2). The gene Glyma04g37040, which encodes a calmodulin-binding protein CML38, was the most induced gene by drought and its transcript levels were induced 40- and 15-fold in R2 and V6* trifolia*, respectively. The most repressed gene by drought was Glyma02g10620, encoding a 98-residue protein of unknown function whose transcript levels were repressed 44- and 4-fold in V6 and R2* trifolia*, respectively.

### 3.4. Common Stress-Responsive MRP-Coding Genes in* Arabidopsis* and Soybean

**Figure 2:**
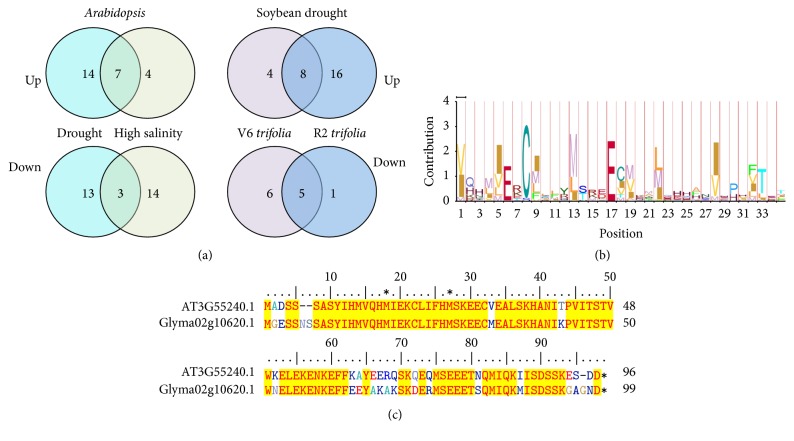
Venn diagram analyses of the expression of MRP-coding genes in* Arabidopsis* and soybean under abiotic stresses (a), and HMM profile of* Arabidopsis* and soybean homologs share common responsiveness to drought (b) and their peptide sequence alignment (c).

In* Arabidopsis*, 7 AtMRPs were upregulated and 3 other AtMRPs were downregulated under both drought and high salinity. In soybean drought, 8 and 5 GmMRPs were up- or downregulated, respectively, in both V6 and R2* trifolia* (Figure 2(a)). Among 6* AtMRPs* encoding calcium signaling proteins, three genes were transcriptionally responsive to either drought or salt (Table S1). At the same time, 14* GmMRPs* encoding calmodulin-like proteins were transcriptionally responsive to drought in either V6 or R2* trifolia* or both. This data suggested that calcium signaling plays an important role in the plant's signaling during abiotic stress exposure. In light of previous studies, it is very likely that Met oxidation and reduction of calmodulin may also contribute significantly to the plant's signaling response to abiotic stresses. A number of* AtMRPs* and* GmMRPs* encoding transcription factors were also responsive to abiotic stresses in* Arabidopsis* and soybean (Tables 1 and 2), indicating the involvement of the MRPs in the important cellular activities.

**Figure 3:**
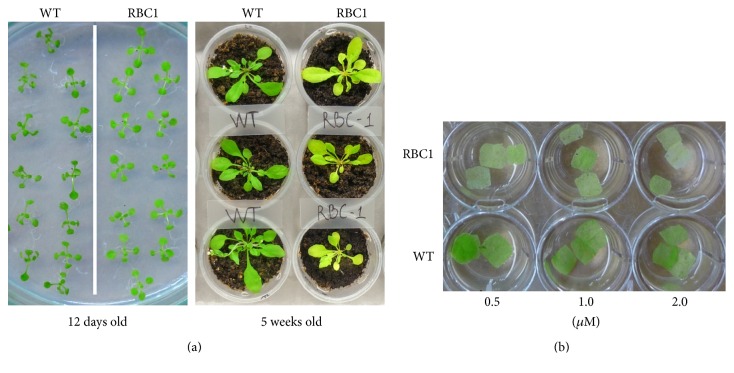
Phenotypes of an* Arabidopsis* overexpressed a gene encoding a MRP under normal physiological condition (a) and under a paraquat leaf disc assay (b). WT, wild type control; RBC1,* Arabidopsis* plant overexpressed At3g55240 gene.

Further analysis of stress-responsive MRP-coding genes identified a gene coding for a plant-specific protein that has homologs in both* Arabidopsis* (AT3G55240) and soybean (Glyma02g10620). These genes encoded highly homologous proteins (>70% identity) of about 100 amino acid residues that share an HMM profile with several conserved Met residues in the N-terminal (Figures 2(b) and 2(c)). In a previous study, it was found that overexpression of this gene in* Arabidopsis* caused a phenotype of pseudo-etiolation in light or leaf bleaching [30], although how such phenotype occurred was not explained. When* Arabidopsis* was transformed with an RNAi construct to downregulate this gene, most of the transgenic plants died at a very early stage and the plants that survived did not show any reduction in the transcription levels, suggesting a vital function of this gene [30]. To determine if the gene At3g55240 is involved in redox stress responses, we acquired the overexpressor line from the* Arabidopsis* FOX line library (RIKEN BioResource Center) and analyzed them. Growing of the plants on MS media and soil confirmed the pseudo-etiolation phenotype (Figure 3(a)). When treated with paraquat in a leaf disc assay, the overexpressor line exhibited higher sensitivity than the wild type control (Figure 3(b)), suggesting the gene may be involved in mediating redox stress responses. This gene and its soybean homolog were both repressed under abiotic stress; thus, increasing its expression may not provide benefit under stress.

### 3.5. Stress-Responsive* cis*-Elements of the MRPs' Promoter

To provide further evidence of the stress-responsiveness of MRP-coding genes, we searched for the presence of known stress-responsive* cis*-elements in the promoters of the genes in* Arabidopsis* 1 kbs upstream of the transcriptional start sites. We found that promoters of* AtMRPs* contain 23, 26, and 16* cis*-elements of ABRE, MYBR, and MYCR, respectively. On average there are 0.54* cis*-elements per AtMRP and 0.86* cis*-elements for each stress-responsive* AtMRP*, indicating a slight enrichment of stress-responsive* cis*-elements among drought- and/or salt-responsive* AtMRPs*.

### 3.6. Subcellular Localization of MRPs

Chloroplast and mitochondria are the two types of cellular organelles which generate high levels of ROS. Thus, identification of MRPs localizing to these organelles may shed light on their functions. Using amino acid sequences of AtMRPs and the prediction tools such as TargetP [37], pSORT [38], and CELLO [39], we identified all AtMRPs targeted to either chloroplast or mitochondria (Table S1). Among 121 AtMRPs, 21 were predicted to target chloroplast by either ChloroP or pSORT or both; 9 were predicted to localize to mitochondria by either pSORT or CELLO or both.

In conclusion, as a complementary approach to other studies on the identification of targets of Met oxidation and reduction in plants, here we found a large number of MRPs involved in important cellular processes such as RNA transcription control and calcium signaling. Several genes encoding these MRPs were transcriptionally responsive to abiotic stresses, such as drought and high salinity, suggesting their roles in the adaptation of plants to these stressors. The fact that promoters of the genes encoding stress-responsive MRPs are slightly enriched in* cis*-element responsive to stresses and that product of these genes were predicted to localize in ROS-enriched organelles, chloroplast and mitochondria, further confirm their functions. Taken together, this work proposes unique evidence on methionine oxidation in proteins and its possible role in regulating the plant's activities.

## Supplementary Material

Met-rich proteins encoded by Arabidopsis (Table S1) and soybean (Table S2) genomes and their transcriptional responses to stress.

## Figures and Tables

**Table 1:** Genes encoding AtMRPs whose expression levels were responsive to both drought and high salinity.

Number	Locus IDs	Met (%)	Length (a.a.)	Drought versus untreated^1^		Salinity versus untreated^2^		Gene descriptions
				Fold change^3^	*q*-value	Fold change^3^	*q*-value	
1	AT1G32560	6.02	134	135.33	0.002	3.31	0.005	LEA group 1 domain-containing protein
2	AT1G33860	8.55	153	2.37	0.092	2.16	0.003	Unknown protein
3	AT3G55240	6.12	95	−60.29	0.007	−26.88	0.001	Overexpression leads to pseudo-etiolation in light phenotype
4	AT3G59900	6.20	130	10.70	0.011	−2.57	0.015	(ARGOS); unknown protein [AT3G59900.1]
5	AT3G62090	6.38	346	64.56	0.020	2.28	0.002	PHYTOCHROME INTERACTING FACTOR 3-LIKE 2
6	AT4G12334	6.25	113	−9.79	0.003	−3.04	0.005	Pseudogene of cytochrome P450 family protein
7	AT4G33467	8.91	102	337.51	0.002	6.16	0.023	Unknown protein [AT4G33467.1]
8	AT4G34590	6.33	159	8.26	0.004	3.27	0.002	GBF6 (*A. thaliana* BASIC LEUCINE-ZIPPER 11)
9	AT5G42325	6.03	233	2.70	0.028	2.45	0.049	Transcription elongation factor-related
10	AT5G67390	7.43	176	−4.17	0.015	−4.15	0.001	Similar to unknown proteins (TAIR:AT1G69360.1)

^1^Two-week-old plants were transferred to soil and allowed to grow for an additional week; the plants were then withheld from watering for 10 days. After 10 d of water with holding, rosette leaves were collected from both well watered and drought-stressed plants in three biological replicates, frozen in liquid nitrogen, and stored at −80°C until used for RNA extraction [35]. ^2^10-day-old plants grown on GM media were transferred onto 0.5 × MS plates without sucrose, containing either 0 mM (control) or 200 mM NaCl, and maintained for a period of 24 h. Three independent experiments were performed for each condition. The samples were collected as three biological replicates (10 plants/replicate), frozen in liquid nitrogen, and stored at −80°C until used for RNA extraction [34]. ^3^Stress responsive AtMRPs were defined as genes encoding MRP whose expression levels were induced or repressed 2-fold or more with an FDR corrected *p* value of less than 0.05.

**Table 2:** Genes encoding GmMRPs whose expression is responsive to drought stress in V6 and R2 leaves.

Number	Glyma ID	Met	Length (a.a.)	V6 *trifolia*		R2 *trifolia*		Gene descriptions	Arabidopsis homologs
		(%)		Fold change	*q*-value	Fold change	*q*-value		
1	Glyma01g15910	8.08	100	3.63	0.045	4.96	0.042	No original description	
2	Glyma01g15930	6.56	458	−20.34	0.007	−3.87	0.015	UNE10; transcription factor	AT4G00050
3	Glyma02g10620	7.22	98	−44.63	0.007	−4.04	0.053	Overexpression leads to pseudo-etiolation in light	AT3G55240
4	Glyma03g32740	6.04	481	−2.19	0.007	−2.02	0.030	PIF1, PIL5; transcription factor	AT2G20180
5	Glyma04g37040	7.91	140	15.03	0.012	40.08	0.005	Calmodulin-binding protein CML38	AT1G76650
6	Glyma06g39910	10.34	117	3.12	0.067	4.14	0.013	Calcium-binding EF hand family protein	AT4G27280
7	Glyma10g30380	7.43	149	7.53	0.013	5.27	0.026	calmodulin 5; calcium ion binding	AT2G27030
8	Glyma15g05510	7.37	96	2.93	0.025	2.41	0.023	No original description	
9	Glyma16g02510	7.26	125	2.05	0.028	4.63	0.023	Calcium-binding protein, putative	AT2G46600
10	Glyma19g43580	6.7	210	−2.01	0.160	2.42	0.078	GIF, GIF1, AN3 (ANGUSITFOLIA3)	AT5G28640
11	Glyma20g00780	6.69	285	−3.03	0.046	−2.36	0.027	Contains homeodomain (InterPro:IPR009057)	AT1G10820
12	Glyma20g22280	6.59	426	2.25	0.046	2.99	0.056	PIF3, POC1, PAP3, transcription factor	AT1G09530
13	Glyma20g36730	7.89	153	3.06	0.042	2.29	0.129	calmodulin 5; calcium ion binding	AT2G27030
